# Ru-induced electronic structure modulation of NiSnSe@GO boosted oxygen and hydrogen evolution reaction under alkaline conditions

**DOI:** 10.1039/d6ra02938h

**Published:** 2026-07-03

**Authors:** Afifah Jabeen, Arif Nazir, Shahid Bashir, Munawar Iqbal, Muhammad Owais, Hassan Tariq, Murat Kaleli, Salih Akyürekli, Abid Ali

**Affiliations:** a Department of Chemistry, The University of Lahore Lahore Pakistan anmalik77@gmail.com; b Higher Institution Centre of Excellence (HICoE), UM Power Energy Dedicated Advanced Centre (UMPEDAC), Level 4, Wisma R&D, Universiti Malaya, Jalan Pantai Baharu Kuala Lumpur 59990 Malaysia; c Zero Emission Technologies Innovation Center, University of Tabuk Tabuk 47913 Saudi Arabia; d Department of Chemistry, Faculty of Science, University of Tabuk Tabuk 47913 Saudi Arabia; e Flexible Electronic Lab, Department of Physics, International Islamic University Islamabad Pakistan; f Innovative Technologies Application and Research Center (YETEM), Süleyman Demirel University Isparta 32260 Turkey

## Abstract

The development of efficient bi-functional electro-catalysts for overall water splitting is crucial for sustainable hydrogen production. In this study, Ru-induced electronic structure modulation is employed to enhance the bi-functional performance of a NiSnSe@graphene oxide (GO) nanocomposite. Ru-NiSnSe@GO catalysts with varying Ru dopant concentrations (0.1%, 0.5%, and 1%) were synthesized *via* a hydrothermal route, followed by electrode fabrication through drop-casting the catalyst ink onto nickel foam. Structural and interfacial coupling were verified by SEM/EDS and elemental mapping (homogeneous distribution of Ni, Sn, Se with dispersed Ru on GO sheets), while XRD peak shifts to higher 2*θ* and Raman band shifts (including G-band red-shift from 1572 to 1565 cm^−1^ at higher Ru loading) evidenced lattice contraction and enhanced charge transfer between the selenide phase and GO. Electrochemical evaluation in alkaline media demonstrated a clear dopant-dependent improvement, with the 1% Ru-NiSnSe@GO exhibiting the best activity with an OER overpotential of 290 mV at 50 mA cm^−2^ with a reduced Tafel slope of 95 mV dec^−1^, and an HER overpotential of 210 mV at 10 mA cm^−2^ with a Tafel slope of 153 mV dec^−1^. The optimized catalyst also showed increased electrochemically active surface area (*C*_dl_ = 0.850 mF; ECSA = 21.25 cm^2^) and markedly lower charge-transfer resistance (*R*_ct_ ≈ 3.2 Ω), supporting faster interfacial kinetics. Overall, synergistic integration of Ru doping with a GO-supported NiSnSe framework provides a practical strategy to boost alkaline OER/HER performance through concurrent active-site enrichment, electronic modulation, and improved conductivity.

## Abstract 2

Ru doping modulates inducing lattice contraction and enhanced charge transfer in NiSnSe@GO. Synergistic Ru–GO interaction boosts kinetics, delivering higher ECSA, reduced charge-transfer resistance and exhibits superior bi-functional performance.

## Graphical abstract

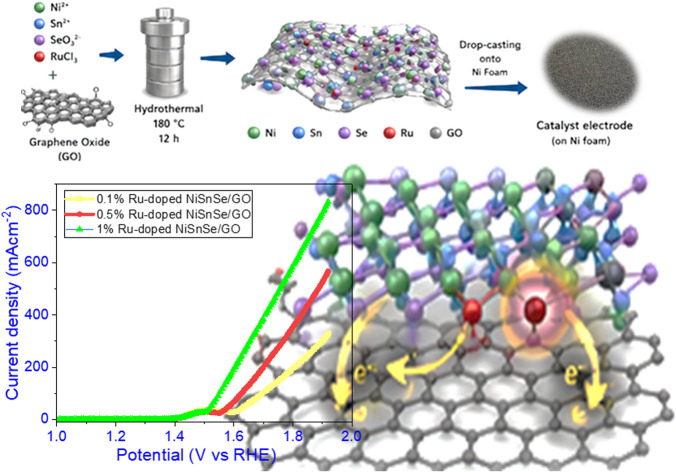

## 1 Introduction

The increasing global energy crisis, compounded by the severe environmental impacts of fossil fuel consumption, has demanded an urgent transition towards sustainable and carbon-free energy systems.^1^ In this situation, hydrogen energy has emerged as a potential alternative due to its high energy density and zero-carbon emission profile upon combustion.^2,3^ Electrochemical water splitting represents one of the most suitable and eco-friendly pathways for high-purity hydrogen production. However, the overall efficiency of water electrolysis is strictly controlled by the thermodynamics and kinetics of its two half-reactions: the hydrogen evolution reaction (HER) and the oxygen evolution reaction (OER).^4,5^ While the HER is a relatively fast process as it involves two electron transfers, the anodic OER involves a complex, thermodynamically uphill four-electron proton-coupled transfer step.^6–8^ This sluggish inherent kinetics results in a large overpotential, thereby acting as the primary bottleneck that hampers the widespread commercialization of electrochemical water splitting technologies.^9^

One of the main approaches to eliminating these bottlenecks is a rational design of active and stable electro-catalysts for both OER and HER. The state-of-the-art OER catalysts in acidic environments and oxy-alkaline environments are precious-metal oxides such as RuO_2_ and IrO_2_, which are typically considered benchmarks due to their favorable adsorption energetics and high inherent activity.^10–13^ However, scarcity and cost motivate intensive research into earth-abundant alternatives.^14–17^ Therefore, much progress has been documented using a wide variety of catalysts including transition-metal oxides (*e.g.*, Ni/Co/Fe oxides and spinels),^18–20^ phosphides (Ni_2_P, CoP),^21,22^ sulfides (MoS_2_, Ni_3_S_2_),^23–25^ layered double hydroxide (LDHs; in particular NiFe-LDH),^26–28^ and selenides (Ni_2_Se, CoSe_2_, multi-metallic selenides).^29–32^ These materials provide adjustable electronic structure, high levels of catalytically accessible sites and opportunities for defect engineering or hetero-structure formation which helps in reducing OER/HER over-potentials and improving long-term stability.

Among these classes, selenium (Se)-based electro-catalysts have received special interest since Se is capable of providing superior electronic polarizability, low electronegativity, as well as discrete metal-chalcogen co-valency as compared to O or S, often enabling improved charge transfer and optimized adsorption energies for key intermediates.^33^ Selenium effectively regulates the electronic structure of the metal centers and results in lowering the overpotential.^34,35^ Furthermore, the catalytic activity of the transition metal selenides is enhanced by the hybridization of Se ‘p’ orbitals and metal's ‘d’ orbital.^36^ These properties of Se not only lower the overpotential required for OER and HER but also provide better stability under alkaline conditions.

In this scenario, Ni–Sn–Se (NiSnSe) based catalysts can offer a promising multi-metallic platform. Ni is well known as a powerful active site in alkaline water splitting particularly where electronic environment is adjusted to stabilize the adsorption of OH^−^/H* intermediates and stabilize oxyhydroxide-like active sites under anodic polarization.^37,38^ Sn, conversely, is frequently used as an electronic and structural modulator, able to tune the properties of the d orbitals of the nearby transition metals. These properties on Sn helps to affect surface hydroxylation, to alter adsorption energetics, and even to stabilize systems more effectively, by inhibiting the unfavorable reconstruction pathways.^39,40^ Consequently, incorporation of Ni and Sn into a Se-coordinated lattice can be used to form active motifs with synergies leading to better catalytic kinetics than single-metal selenides.

The practical bi-functionality of binary nickel selenides is usually compromised by suboptimal hydrogen binding energies, whereas the OER activity is promising. In this work, Ni–Se lattice was employed as a host lattice for the strategic incorporation of Sn as an electronic and structural modulator. Lattice strain and redistribution of electron density around the Ni centers brought about by the difference in the atomic radius and electronegativity of Sn optimize the adsorption energies for HER and OER intermediates. This particular ternary NiSnSe matrix enables one to achieve maximum catalytic activity with minimum amount (1%) of noble Ru dopant.

In addition to composition, active area and transport properties are highly controlled by the catalyst support as well as nano-architecture. Graphene oxide (GO) has commonly been used in electro-catalysis since it provides a high surface area scaffold, sufficient oxygenated functional groups (epoxy, hydroxyl, carboxyl) to facilitate nucleation/anchoring of nano-phases and the possibility to create percolative conductive networks (especially through partial reduction during processing or electrochemical activity).^41^ Despite being less conductive than graphene, the defect chemistry of pristine GO and its interfaces can be utilized to improve dispersion, inhibit agglomeration, and improve electrode integrity, all of which are important to high current density stable operation.^42^ Direct growth of NiSnSe onto GO can thus be beneficial as ultra-small, well-dispersed domains can maximize exposure of Ni/Sn active domains, strong interfacial coupling that enhances electron-transport of catalytic sites to current collector and can be porous enough to enable mass-transport applications. The given hybridization strategies of anchoring chalcogenide catalysts on graphene-based supports are highly viable routes in enhancing activity, stability, and practical electrode utilization.^43^

Finally, the doping of Ru into earth-abundant electro-catalysts is also being considered to fill the performance gap between cheap catalysts and noble-metal oxides. Trace Ru incorporation can be used in multicomponent chalcogenide/graphene systems in distinct ways, as an electrochemical modulator, where Ru changes the local charge density and optimizes adsorption energies of OER/HER intermediates (*e.g.*, OH*, O*, OOH* or H*), as a structural modulator, where Ru can cause lattice strain, defect formation (*e.g.*, vacancies), and altered coordination environments to generate more catalytically competent sites, and as an interfacial activator, where Ru may improve charge-transfer kinetics across NiSnSe–GO boundaries and promote favorable surface reconstruction under OER potentials.^44–47^

This study investigates the electro-catalytic modulation of transition metal selenides@GO nanocomposites through ruthenium doping, aiming to optimize their performance for the oxygen evolution reaction by exploiting synergistic effects and enhanced charge transfer. This approach leverages the exceptional conductivity of graphene oxide as a scaffold to mitigate the agglomeration of metal selenides, thereby improving electron and mass transfer, and creating a robust platform for efficient OER. This synergistic integration of ruthenium-doped transition metal selenides with graphene oxide aims to unlock new avenues for developing highly active and stable electro-catalysts for industrial-scale water splitting.

## 2 Experimental section

### 2.1 Chemicals and materials

All chemicals were of analytical grade and used without further purification. Ethanol (C_2_H_5_OH, 99.8%), tin chloride dihydrate (SnCl_2_·2H_2_O, 99.9%), nickel chloride hexahydrate (NiCl_2_·6H_2_O, 99.8%), potassium hydroxide (KOH, 99.9%), graphene powder (98%), selenium powder (99%) and hydrazine monohydrate (N_2_H_4_·H_2_O, 99%) were purchased from Sigma/Merck. In all experiments, double distilled water was used.

### 2.2 Preparation of Ru-doped TMSe@GO

**Scheme 1:**
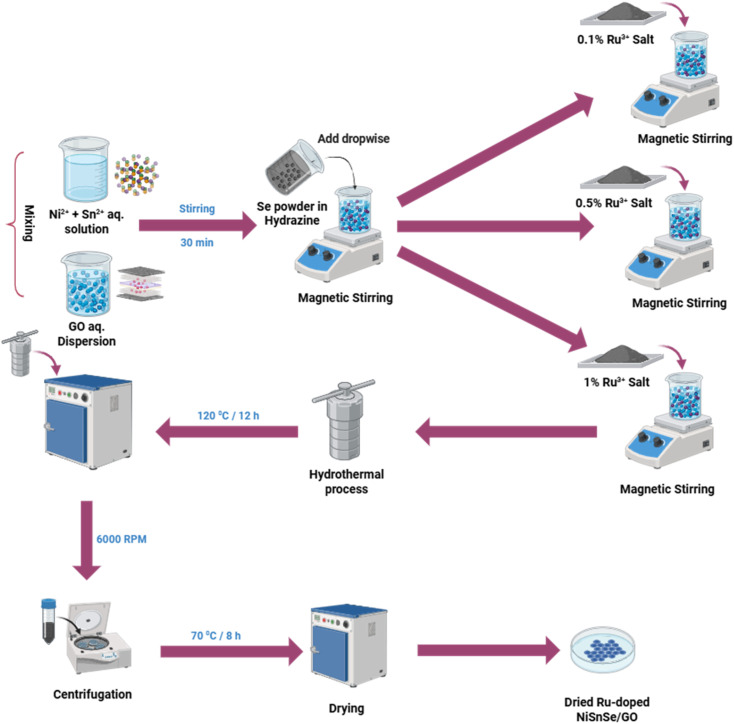
Schematic illustration for the synthesis of Ru-doped NiSnSe@GO.

Ru-doped TMSe@GO was synthesized using a previously reported procedure as illustrated schematically in Scheme 1. A mixed metal-salt solution was prepared by dissolving 30 mM NiCl_2_·6H_2_O and 30 mM SnCl_2_·2H_2_O (Ni : Sn = 1 : 1) in 150 mL of distilled water. Separately, 45 mg of GO was dispersed in 60 mL of distilled water, added dropwise to the metal-salt solution, and stirred for 30 min. In parallel, a stable selenium suspension was prepared by immersing 60 mg of Se powder in 60 mL of hydrazine hydrate. This Se suspension was then added dropwise to the NiSn@GO mixture and vigorously stirred for 1 h.

The resulting TMSe@GO dispersion was divided into three equal portions, and Ru was introduced of a Ru precursor salt (RuCl_3_·*x*H_2_O) by adding 0.1%, 0.5%, and 1% separately to each portion. Each mixture was then transferred into a 100 mL autoclave and hydrothermally treated at 120 °C for 12 h, followed by natural cooling to room temperature. The obtained composite was collected by centrifugation at 6000 rpm, washed with distilled water, and dried at 70 °C for 8 h. The same procedure was repeated for the remaining two portions to obtain the corresponding Ru-doped materials.

### 2.3 Fabrication of electrode

**Scheme 2:**
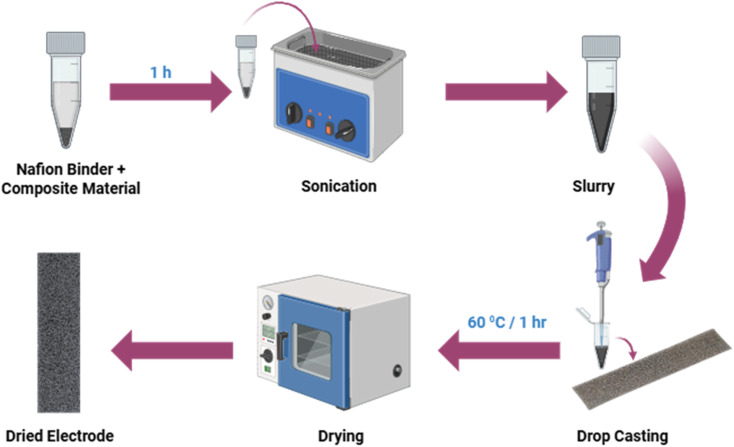
Schematic illustration of the electrode fabrication *via* drop casting method.

Nickel foam (NF) was used as the substrate to evaluate the electrochemical activity of the Ru-doped TMSe@GO composite. NF pieces (1 × 1 cm^2^) were cut and pretreated to remove the surface oxide layer by immersion in a cleaning solution containing distilled water, ethanol, and sulfuric acid. For electrode fabrication, an ink/slurry was prepared by dispersing 5 mg of the composite in 1 mL of a mixed solvent containing Nafion binder, ethanol, and water. The resulting slurry was drop-cast onto the pretreated NF using a micropipette, as illustrated in Scheme 2. The coated electrodes were then dried at 60 °C for 6 h and subsequently used for OER and HER measurements.

### 2.4 Physical characterization

Rigaku Ultima IV powder X-ray diffractometer with Cu Kα line was used to acquire XRD patterns to study the crystal phase and nanostructure of the synthesized Ru-doped TMSe@GO samples. The morphology of the composite material was determined by obtaining the scanning electron microscope (SEM) images at different resolution by using Bruker D8 Advance Twin–Twin (40 kV, 40 mA) Cu Kα radiation (*λ* = 1.5418 Å) scanning electron microscope (SEM) brand model: FEI Quanta FEG 250. Raman spectroscopy has been analyzed *via* RAMAN: WITec alpha 300R.

### 2.5 Electrochemical characterization

Gamry Reference 6000 Potentiostat/Galvanostat was used to measure all OER activities using three electrode system. Ru-doped TMSe@GO modified NF was used as working electrode and Ag/AgCl and platinum sheet was used as reference and counter electrodes. 1 M KOH aqueous solution was used as an electrolyte. Linear sweep voltammetry (LSV) curves were obtained in the potential window of 0 to 1 V (*vs.* Ag/AgCl) reference electrode at a scan rate of 5 mV sec^−1^. This potential was then converted to reversible hydrogen electrode (RHE) value by using the following eqn (1) given below.^48^

1 *E*_RHE_ = *E*_Ag/AgCl_ + *E*_0_ + (0.059 × pH)

Kinetics of the reaction was studied by plotting Tafel slope using the LSV data. Eqn (2) helps to study the linear Tafel slope.2
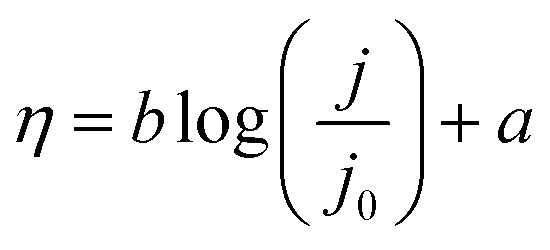
where *η* is the overpotential, *j*_0_ is exchange current density, *j* is current density, *b* is Tafel slope and ‘*a*’ is a constant associated with the activation energy of the reaction. Double layer capacitance (*C*_dl_) of the electrode material was calculated by using CV curves. The electrochemically active surface area (ECSA) was calculated from *C*_dl_ value. Electrochemical impedance spectroscopy (EIS) was applied to calculate impedance measurements using 1 M KOH aqueous electrolyte solution. For OER and HER with DC voltage of 0.65 V, the frequency range was set from 0.1 Hz to 1 MHz.

The polarization curves of all linear sweep voltammetry (LSV) and reported over-potentials were iR-corrected to remove the ohmic voltage drop (due to the electrolyte resistance). The uncompensated solution resistance (*R*_s_) for every measurement was obtained from the respective Electrochemical Impedance Spectroscopy (EIS) Nyquist plots. The potentials were corrected manually (or automatically by the potentiostat) with *E*_corrected_ = *E*_measured_ − *I* × *R*_s_ to ensure that the potentials reported are the real intrinsic catalytic activities of the fabricated electrodes.

## 3 Results and discussion

### 3.1 Physical characterization

#### 3.1.1 Scanning electron microscopy (SEM)

**Fig. 1:**
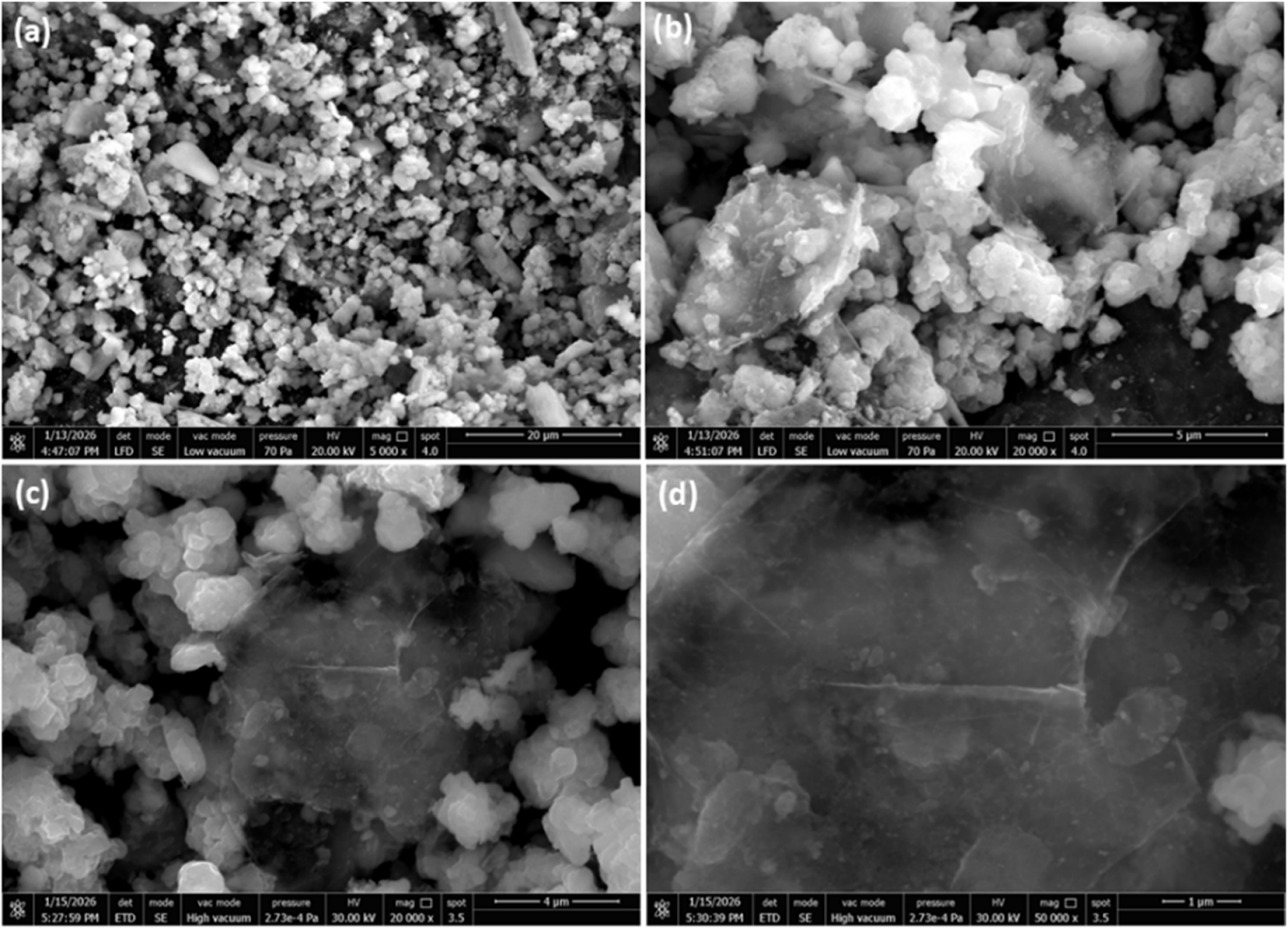
SEM micrograph of the synthesized 0.1% Ru-NiSnSe@GO at (a and b) lower and (c and d) higher magnification.

Scanning electron microscopy (SEM) is used to study the surface morphology of the synthesized nanocomposites to provide direct information about the microstructural features of the nanocomposites, the uniformity and aggregation behavior and textural characteristics.^49^ The SEM and EDS results collectively prove the successful fabrication of a 0.1% Ru-NiSnSe@GO composite featuring a hierarchical, rough and porous architecture, in which the active chalcogenide phase is successfully embedded within the GO support, as shown in Fig. 1.^50^ The morphological structure reveals wrinkled sheet-like GO domains, which can form a conductive and mechanically robust scaffold while granular NiSnSe features are distributed well across the sheets, creating a richly exposed surface area and interconnected voids, capable of allowing electrolyte penetration and efficient gas-bubble release during electro-catalysis. The corresponding EDS spectrum confirms the existence of Ni, Sn, and Se, along with C and O from GO, and additionally, a perceivable Ru contribution, which is in line with the successful incorporation of trace amounts of Ru. This synthesized composite material seems to indicate a strong catalyst-support coupling, good conditions for improved charge transfer and active sites accessibility.

**Fig. 2:**
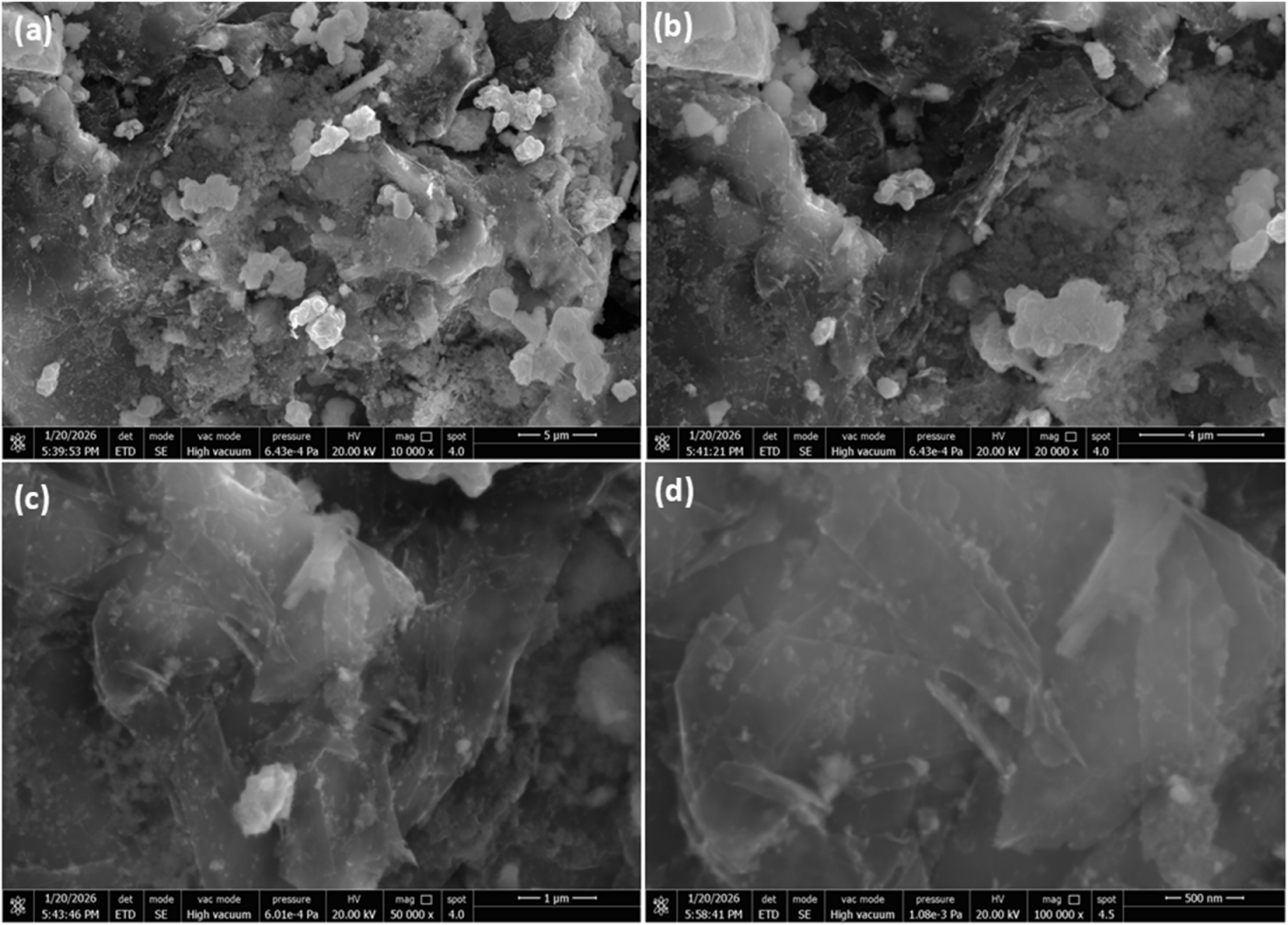
SEM micrograph of the synthesized 0.5% Ru-NiSnSe@GO at (a and b) lower and (c and d) higher magnification.

For the 0.5% Ru-NiSnSe@GO sample the SEM micrographs is shown in Fig. 2a–d and EDS spectrum is shown in Fig. 4a. The results show the formation of a sheet-based scaffold (attributed to GO), uniformly decorated with granular/clusters of inorganic domains, which confirms the studding of the NiSnSe phase on the surface of the GO, rather than being comprised of a separated bulk phase. This results in a rough, heterogeneous and porous texture which is expected to increase accessible surface area, expose more of the catalytic sites and provide open pathways for electrolyte transport and gas evolution under the conditions of HER and OER. EDS further supports this interpretation confirming Ni, Sn and Se to be present along with C and O and Ru showing peaks consistent with noble metal incorporation, still at this intermediate loading Ru is expected to be largely dispersed on the surface and at interfaces to promote better charge transport and improved catalytic kinetics.

**Fig. 3:**
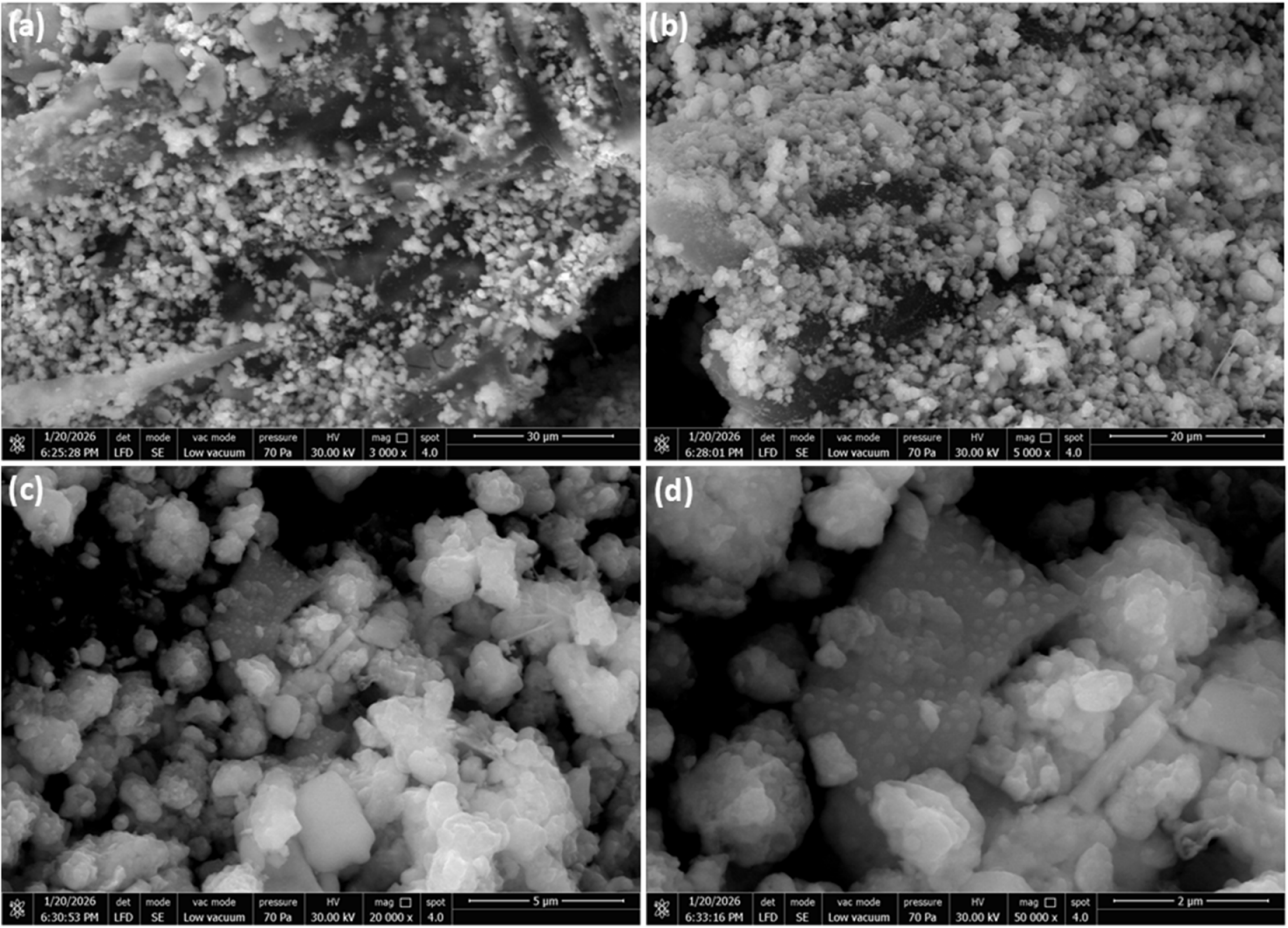
SEM micrograph of the synthesized 1% Ru-NiSnSe@GO at (a and b) lower and (c and d) higher magnification.

In the case of 1% Ru-NiSnSe@GO, SEM shown in Fig. 3 that the coating consists of rough particles anchored on sheet-like GO domains, showing good incorporation of the active chalcogenide phase into the conducting carbon scaffold. The granular and interconnected network that is formed is anticipated to contribute to enhanced electro-catalytic behavior. This improved catalytic property is due to the increased accessible surface area, improved electrical percolation throughout the catalyst layer, as well as facilitated access to electrolyte and release of gases during operation. In agreement with this, EDS validates the elements composition by showing characteristic signals of Ni, Sn and Se in addition to C and O from GO as well as showing distinct Ru peaks validating the successful incorporation at the 1% level. Overall, these structural and compositional features demonstrate the good formation of Ru-NiSnSe@GO composite with favorable properties for efficient water splitting electro-catalysis.

**Fig. 4:**
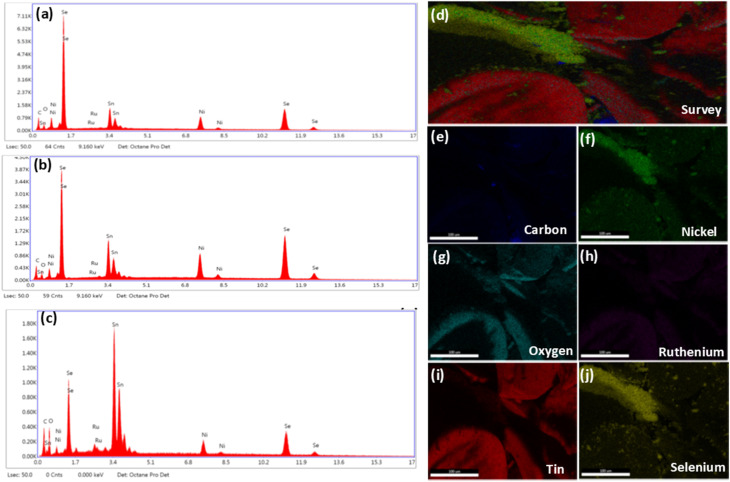
EDS analysis of synthesized 0.1% Ru-NiSnSe@GO (a), 0.5% Ru-NiSnSe@GO (b) and 1% Ru-NiSnSe@GO (c). Elemental mapping analysis showing (d) survey analysis and the elemental form of (e) carbon, (f) nickel, (g) oxygen, (h) ruthenium, (i) tin and (j) selenium.

#### 3.1.2 Elemental mapping

Elemental mapping of 1% Ru-NiSnSe@GO composite is shown in Fig. 4d–j, which confirms the successful incorporation of the active phase with the GO support phase. The C in Fig. 4e and O in Fig. 4g maps indicate the presence of a continuous GO framework that possesses oxygen containing functional groups, which provide several anchoring sites for inorganic components. The Ni in Fig. 4f, Sn in Fig. 4i, and Se in Fig. 4j maps show good spatial overlap that confirms the formation of a well distributed Ni–Sn–Se chalcogenide phase on the GO sheets with no notable segregation of the elements. The Ru map in Fig. 4h shows a weaker, but clearly detectable, signal that is spread throughout similar regions of the NiSnSe phase, suggesting successful incorporation of Ru at 1% loading without formation of large clusters of Ru-rich phase. Overall, the observed co-localization and homogeneous dispersion facilitate strong interfacial contact and homogeneous distribution of the active sites, which are favorable for the efficient charge transfer and enhanced electro-catalytic performance.^51^

EDS, elemental mapping and XRD analysis are used to confirm the successful synthesis and uniform integration of Ru-NiSnSe@GO composite. Elemental mapping (Fig. 4h) shows that Ru was uniformly dispersed with Ni, Sn and Se, which means there is no segregated phase or isolated clusters of Ru. EDS spectra definitely confirm the presence of Ru. In addition, the XRD diffraction peaks of the successfully incorporated Ru indicated lattice alteration by showing distinct shifts from the standard diffraction pattern (JCPDS # 00-011-0552), which was not a physical mixture. Future high resolution surface studies (TEM/XPS) will be useful in mapping atomic level boundaries, but these combined structural and functional parameters are sufficient to demonstrate the successful preparation of the highly active Ru-doped composite.

#### 3.1.3 X-ray diffraction spectroscopy (XRD)

**Fig. 5:**
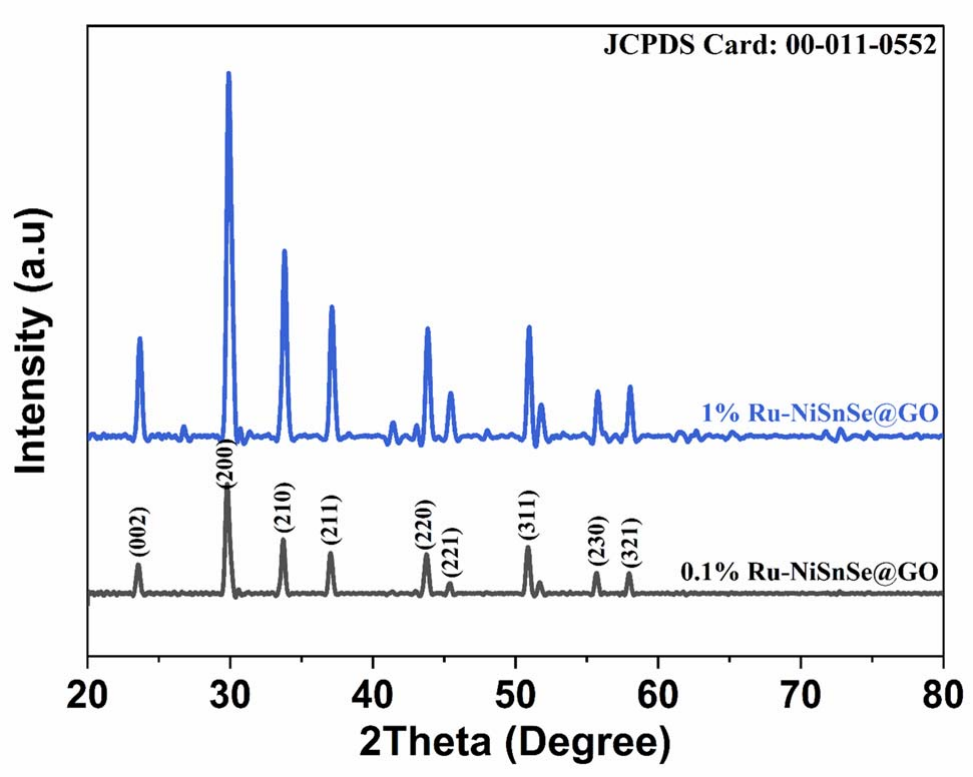
X-ray diffraction pattern of synthesized 0.1% Ru-NiSnSe@GO and 1% Ru-NiSnSe@GO.

X-ray diffraction (XRD) analysis was employed as a primary non-destructive technique to definitively elucidate the crystallographic structure, phase purity, and structural integrity of the synthesized nanomaterials. By adhering to Bragg's Law (*nλ* = 2*d* sin *θ*), the diffraction patterns provide critical insights into the lattice dimensions and atomic arrangements within the host matrix. Fig. 5 shows the X-ray diffraction (XRD) pattern of the obtained nanocomposite Ru-NiSnSe@GO. The diffraction profile shows strong peaks of high intensity, which confirms the high crystallinity of the material. All of the observed reflection peaks can be well-indexed based on the cubic pyrite structure (space group: *Pa*3̄) of NiSe_2_, which is in accordance with the JCPDS Card no. 00-011-0552.^52,53^ The characteristic diffraction peaks which are present at a 2*θ* value of about 29.9°, 33.7°, 37.1°, 43.80°, 45.42°, 50.93°, 55.61° and 58.03° correspond to the (200), (210), (211), (220), (221), (311), (230) and (321) crystallographic planes respectively. The peak at 23.58° with plane (002) might be attributed to the graphitic carbon (GO or rGO) in the sample.^54^

Interestingly, no distinct diffraction peaks for tin (Sn), tin selenides (SnSe) or metallic ruthenium (Ru) could be detected. The non-observation of these peaks indicates that the Sn and Ru doping have been successfully inserted into the host NiSe_2_ lattice by substitutional doping to form a solid solution without phase segregation.^52^ Furthermore, a very careful comparison with the standard JCPDS data shows a small shift of the diffraction peaks to higher 2*θ* value (to the right). This positive shift suggests that the inter-planar *d*-spacing decreases as explained by Bragg's Law (*nλ* = 2*d* sin *θ*), which can be attributed to contraction of the lattice. This structural contraction provides further evidence for the partial substitution of larger Ni ions by smaller Ru ions into the crystal matrix and further validates the successful doping of the material. The observed lattice contraction and peak shifting confirm the successful incorporation of both Sn and Ru. The atomic size disparity between Sn and Ni induces beneficial structural strain, creating defect-rich sites that serve as highly active catalytic centers compared to un-alloyed binary Ni-selenides.

The systematic shift in the XRD peaks toward higher 2*θ* values provide strong evidence for a decrease in inter-planar *d*-spacing, although direct atomic-scale observation is necessary for absolute confirmation. The uniform change of the unit cell, together with the good electronic coupling seen in Raman measurements, suggests strong and converging evidence for the successful incorporation of the trace Ru in the host NiSnSe lattice as a substitutional dopant, and not just as isolated surface clusters.

#### 3.1.4 Raman spectroscopy

**Fig. 6:**
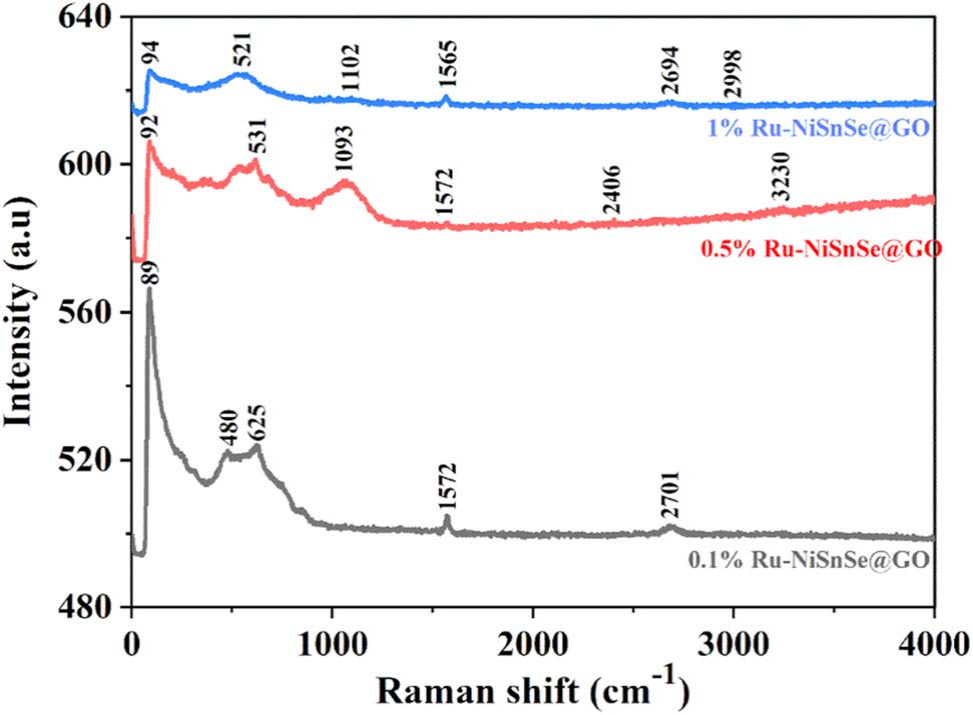
Raman plots demonstrating the functional properties and phase transitions of various Ru-NiSnSe@GO samples.

Raman spectroscopy is a powerful, non-destructive technique which is used here to explore the structural fingerprints, vibrational modes and electronic interactions in the synthesized nanocomposites. Unlike the X-ray diffraction method which yields information on the long-range crystalline order, the Raman spectroscopy technique gives important information on the local structural environment, the defect density and the interfacial charge transfer between the metal selenide species as well as the GO support. Fig. 6 shows the comparative Raman spectra results of 0.1%, 0.5% and 1% Ru-NiSnSe@GO. The spectra show distinct vibrational modes of both the metal-selenide lattice and the carbonaceous support.

In the low-frequency region, a strong peak is observed around 89–94 cm^−1^, which is attributed to characteristic modes of lattice vibration (A_1g_) of the metal selenide (NiSnSe) structure.^53^ Interestingly as the Ruthenium concentration increases from 0.1% till 1% this peak shows a blue shift (from 89–94 cm^−1^). This shift to a higher wavenumber is an indication of a stiffening of the lattice bonds and a contraction in the unit cell, which confirmed the successful incorporation of the Ru atoms into the host lattice, which causes an alteration to the local vibrational energy. In the high frequency region, the characteristic graphitic bands can be seen clearly. The peak in the region of 1572 cm^−1^ is associated to the G-band and is the in-plane stretching vibration of sp^2^ hybridized carbon atoms.^54^

A noticeable phenomenon is noted in the case of the 1% Ru-doped sample, the G-band is red shifted from 1572 cm^−1^ (in the 0.1% sample) to 1565 cm^−1^. This downward shift in the G-band frequency is indicative of n-type doping, implying a significant electron transfer from Ru doped metal selenide nanoparticles to graphene oxide sheets. This is an electronic interaction that promotes better conductivity and the kinetics of charge transfer in electrochemical processes. In addition, the fact that the 2D band (around 2701 cm^−1^ for the 0.1% sample), which also shifts to 2694 cm^−1^ in the 1% sample, is also present is a further support of the modification of the electronic structure of graphene support due to the strong coupling with the catalytically active metal selenide phase.

### 3.2 Electrochemical characterization

#### 3.2.1 Linear sweep voltammetry (LSV)

The generated Ru-NiSnSe@GO nanocomposites' electro-catalytic activities were fully examined using a combination of Tafel slope measurements and Linear Sweep Voltammetry (LSV). These alternate methods are valuable resources for learning about the reaction's kinetics and catalytic activity.^55^

**Fig. 7:**
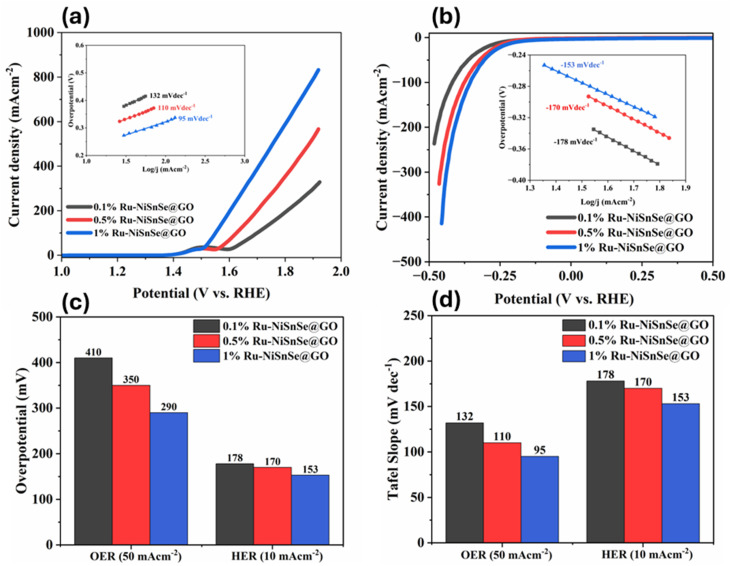
(a) LSV curves for OER with in-set showing Tafel slope, (b) LSV curves for HER with in-set showing Tafel slope, (c) bar-graphs of over-potentials for OER and HER, (d) bar-graphs of Tafel slopes for OER and HER.

Fig. 7a shows LSV curves for OER in the bare NF, commercial CuO_2_, samples of 0.1%, 0.5% and 1% Ru doping. A unique trend in electro-catalytic efficiency is observed along with the increase in concentration of the dopant. With the lowest excessive potential of 290 mV at 50 mA cm^−2^ to initiate the reaction, the 1% Ru-NiSnSe@GO electrode performs better. By contrast, the overpotential values needed for the 0.5% and 0.1% doped samples were greater (350 mV and 410 mV, respectively). This decrease in overpotential with the increment of Ru content suggests the increased presence of active sites accessible for the reaction and therefore charge transfer at the electrode–electrolyte interface, is increased. However, in this study, the maximum concentration of Ru doping was carefully chosen to be 1% to avoid the possibility of phase segregated noble metals and to be within the scope of a low-cost, trace-doping approach to assess the enhancement of the transition metal selenide framework.

The OER activity of bare Nickel Foam (NF) and commercial RuO_2_ were tested as controls to accurately isolate the intrinsic catalytic activity of the synthesized materials and to benchmark the practical viability of the synthesized materials given in Fig. S1 (SI). The bare NF shows low OER activity (overpotential of 500 mV at 10 mA cm^−2^, as shown Fig. 6a) which indicates that the background contribution from the substrate is almost negligible. Moreover, the overpotential of 1% Ru-NiSnSe@GO composite is only 290 mV, much lower compared to commercial RuO_2_ (430 mV). This indicates that the catalytic activity is naturally attributed to the synergistic structural benefits of the composite and is superior to the traditional commercial catalysts.

To further quantify the reaction kinetics, Tafel slope analysis was carried out using the following equation: *η* = *b* log(*j*) + *a*.^8,56^ As seen in Fig. 7a, the 1% Ru doped sample shows the lowest Tafel slope of 95 mV dec^−1^, which suggests that the charge transfer efficiency is higher and the reaction kinetics are faster. In contrast, the values of 110 and 120 mV dec^−1^ were greater in the 0.5% and 0.1% samples. To ensure high activity and electron transfer rate, an effective electro-catalyst should ideally have a low Tafel slope and low overpotential. In the presence of the NiSnSe@GO matrix, the gradual decrease in Tafel slope values demonstrates the efficacy of higher ruthenium doping levels in lowering the activation energy barrier value, which further boosts catalytic intrinsic activity. The presence of Ru in the sample decreases the Tafel slope from 132 mV dec^−1^ (0.1% sample) to 95 mV dec^−1^ (1% Ru-doped sample), suggesting that the incorporation of Ru significantly lowers the activation barrier for the initial adsorption of the OH^−^ intermediate, thereby driving the OER mechanism towards a more efficient kinetic pathway.

The increase in electro-catalytic activity with higher ruthenium concentration can be explained by the mutual influence between electronic modulation and number of active sites available. Firstly, the introduction of an increase in ruthenium (1%) *i.e.* a higher concentration of ruthenium can significantly increase the number of catalytically active sites on the surface of the NiSnSe matrix, providing more centers for the electrochemical reaction to occur. Secondly, as shown by the XRD analysis the doping of substitutional Ru in the host lattice leads to a contraction of the lattice. It is known that this structural distortion modifies the host metal ions' electronic structure (d-band center), which maximizes the adsorption energy of the reaction intermediates and reduces the activation energy barrier. Additionally, the conducting GO network and the presence of ruthenium facilitate the rapid flow of electrons across the electrode–electrolyte interface. The lowest Tafel slope (95 mV dec^−1^ zone recorded for 1% sample), which indicates that the reaction kinetics are greatly accelerated for high doping levels, provides numerical confirmation of this improved charge transfer capabilities.

Fig. 7b shows cathodic polarization curves (HER) of the 0.1%, 0.5% and 1% Ru-NiSnSe@GO electro-catalysts. As shown by the LSV curves, electro-catalytic activity highly depends on the ruthenium doping concentration. The 1% Ru doped exhibits lower overpotential of 210 mV at current density of 10 mA cm^−2^ indicating better HER activity. In contrast, the 0.5% and 0.1% samples need higher over-potentials of 230 mV and 250 mV respectively. This decrease in overpotential (by 40 mV when compared to the 0.1% sample) emphasizes the effectiveness of optimized Ru-doping for the reduction of activation energy barrier for H_2_ production.

In order to further study, the reaction kinetics and the rate-determining step, Tafel slopes were calculated using the linear region of the polarization curves (inset of Fig. 7b). A smaller Tafel slope usually means the reaction is faster. The calculated Tafel slope values are 178 mV dec^−1^, 170 mV dec^−1^ and 153 mV dec^−1^ for the 0.1%, 0.5% and 1% samples respectively. The 1% Ru doped sample shows the lowest Tafel slope, which implies that the hydrogen evolution kinetics are greatly facilitated on its surface. This enhanced performance is attributed to the synergistic effect of the metal selenide with Ru and graphene oxide support, which facilitates electron transfer and optimization of the adsorption energy of hydrogen intermediates at the surface of the catalyst.

For alkaline HER, a Tafel slope larger than 120 mV dec^−1^ quantitatively indicates that the Volmer step (water dissociation: H_2_O + e^−^ → H_ads_ + OH^−^) is the rate-determining step. The initial slope of 178 mV dec^−1^ is large because the H–O–H bond is cleaved slowly on the lightly doped matrix. The reduction to 153 mV dec^−1^ in the 1% Ru doped sample, however, provides a clear demonstration of the mechanism in which the introduced Ru centers are effective at activating water molecules. Ru lowers the energy barrier for the Volmer step and hence, it serves as a catalyst for the formation of adsorbed hydrogen (H_ads_), which in turn has a profound effect on the hydrogen evolution kinetics.

#### 3.2.2 Cyclic voltammetry

**Fig. 8:**
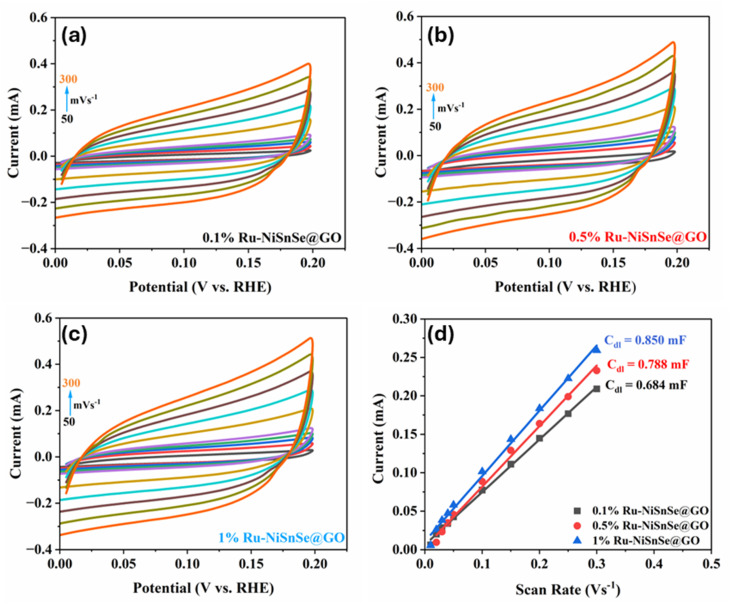
(a–c) CV curves and (d) linear plot of current *vs.* scan rate for 0.1%, 0.5% and 1% Ru-NiSnSe@GO samples.

Cyclic voltammetry (CV) measurements were carried out in the non-faradaic region to ascertain the electrochemically active surface area (ECSA) of synthesized materials and to comprehend the intrinsic nature of improved electro-catalytic actions in the form of double layer capacitance (*C*_dl_). Fig. 8a–c shows the CV curves of the 0.1%, 0.5% and 1% Ru-Ni-SnSe@GO nanocomposites, which were tested in the voltage range of 0.00 to 0.20 V *vs.* RHE at different scan rates from 50 to 300 mV s^−1^. As observed from the CV curves, all three samples show characteristic quasi-rectangular shapes with no sharply defined redox peaks which indicates that the current response in this potential range is controlled by double-layer charging capacitance (*C*_dl_) rather than by faradaic reactions. The voltametric current density for all samples shows a linear growth with increasing scan rates, indicating very good electrochemical stability and the free transport of ions at the electrode–electrolyte boundary.

The *C*_dl_ values, proportional to the ECSA, were determined by plotting the difference in current density 
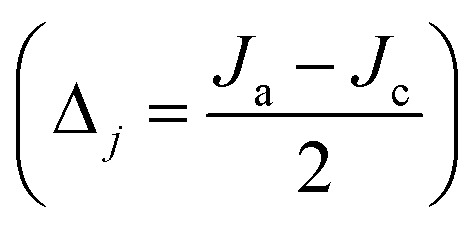
 at 0.10 V *versus* the scan rate as is seen in Fig. 8d. The slope of the linear fit is equal to *C*_dl_ (double-layer capacitance). For samples doped with 0.1%, 0.5%, and 1% Ru, the computed values of *C*_dl_ are 0.684 mF, 0.788 mF, and 0.850 mF, respectively. There is a clear trend: as the concentration of ruthenium rises, the value of *C*_dl_ grows steadily. The 1% Ru doped sample has a high *C*_dl_ value of 0.850 mF, which is very high compared with the values of 0.5% and 0.1%. This increased capacitance represents a larger ECSA and therefore indicates that the optimized Ru-doping not only alters the electronic structure, but also much more catalytically active sites are exposed to the electrolyte. This enhanced accessibility of active sites enables more efficient charge transfer, which positively correlates with the better electro-catalytic activity (lowest overpotential and Tafel slope) for the electrodes found in the LSV analysis.

The electrochemically active surface area (ECSA) was then calculated from the double-layer capacitance *C*_dl_ values to provide a measure of the density of exposed active sites. The ECSA has been estimated by formula ECSA = *C*_dl_/*C*_s_ where *C*_s_ is the specific capacitance of a flat standard electrode and was assumed to be 0.040 mF cm^−2^ for Ni-based materials in alkaline media. Based on the calculated *C*_dl_ values of the samples, the ECSA of the samples with 0.1%, 0.5% and 1% Ru doping was found to be 17.1 cm^2^, 19.7 cm^2^, 21.25 cm^2^, respectively. The largest electrochemically active surface area is shown on the 1% Ru-NiSnSe@GO sample which is about 1.24 times greater than the 0.1% doped sample. This important enlargement in active surface area confirms the effective prevention in agglomerating the nanoparticles by increasing the ruthenium dopant concentration and exposes a greater number of active catalytic sites to the electrolyte to achieve enhanced performance in splitting water.

The activity was carefully studied to ensure that the increased catalytic activity is not due to any possible surface area, loading, or other random effects, but to the intrinsic electronic modulation. The standardized catalyst loading (1.0 mg) automatically resulted in the highest mass activity for the 1% Ru-NiSnSe@GO catalyst. Moreover, the intrinsic activity was assessed using the ECSA obtained from double-layer capacitance (*C*_dl_). The *C*_dl_ of the 1% sample was the highest among the others (0.850 mF), however the significant increase in overpotential (290 mV) and charge transfer resistance (4.6 Ω) was so great that it was masking the surface-area expansion. These ECSA-normalized specific activity values are complemented by the extremely positive OER Tafel slope of 95 mV dec^−1^ which demonstrates that the effective catalytic reaction kinetics of the active sites is improved beyond simply the morphological improvements that result from Ru-doping.

#### 3.2.3 Electrochemical impedance spectroscopy

**Fig. 9:**
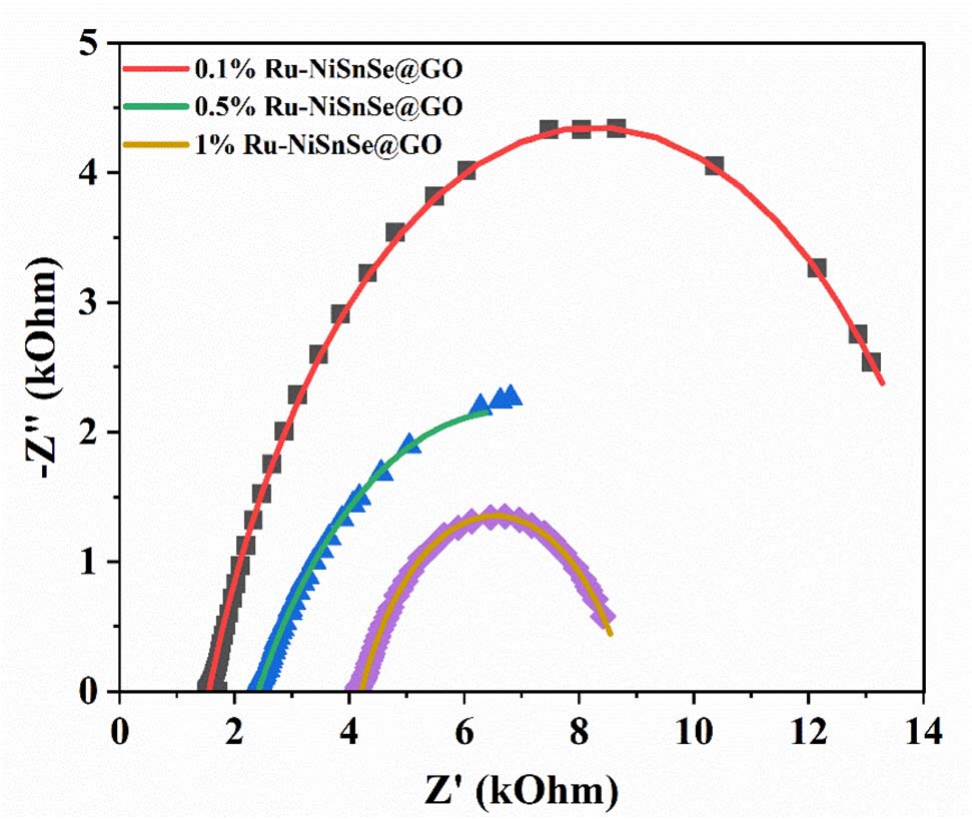
Nyquist plot for 0.1%, 0.5% and 1% Ru-NiSnSe@GO for EIS.

Electrochemical Impedance Spectroscopy (EIS) analysis was carried out at constant current density (50 mA cm^−2^) to measure the electrochemical kinetics during the Oxygen Evolution Reaction (OER). The resulting Nyquist plots as shown in Fig. 9 were fitted with a simple Randles equivalent circuit model [*R*_s_ (CPE‖*R*_ct_)], where the solution resistance (*R*_s_) and a constant phase element (CPE) representing the non-ideal double layer capacitance and the charge transfer resistance (*R*_ct_) are present. The *R*_s_ values of three samples were found to be low and similar (approx. 18–25 ohm) implying consistent electrolyte conduction and good electrode contact in all the measurements. However, a considerable variation in charge transfer resistance (*R*_ct_) which is the dicing determinant with respect to catalytic activity was observed.

The 0.1% Ru-doped sample showed a huge semicircle with high *R*_ct_ value about 13.5 ohm showing a large barrier in the electron transfer at the catalyst–electrolyte interface. On increasing the doping concentration up to 0.5%, it decreased to *R*_ct_ = 7.8 ohm. Most notably, the sample Ru-NiSnSe@GO 1% showed the smallest semicircle diameter with greatly reduced *R*_ct_ value of 3.2 ohm. This result of 4.2 times resistance compared to the 0.1% sample proves that the optimal ruthenium doping has a very large effect on the intrinsic electrical conductivity of the material. This promotes the fast transportation of electrons from the surface of the electrode to the reactants at 50 mA cm^−2^, which promotes the splitting of the water, thus reaction, faster. Different electrochemical parameters for the all fabricated electrode have been summarized in (Table 1).

**Table 1:** LSV data of overpotential and Tafel slope of both OER and HER, CV data of *C*_dl_ and ECSA and EIS data for *R*_s_ and *R*_ct_

Electrocatalyst	HOER	*η* HER	Tafel slope OER	Tafel slope HER	*C*_dl_ (mF)	ECSA (cm^2^)	*R*_s_ (ohm)	*R*_ct_ (ohm)
0.1% Ru-NiSnSe@GO	410	250	132	178	0.684	17.10	1.54	13.42
0.5% Ru-NiSnSe@GO	350	230	110	170	0.788	19.70	2.41	8.66
1% Ru-NiSnSe@GO	290	210	95	153	0.850	21.25	4.20	4.60

#### 3.2.4 Stability test

Ru-doped NiSnSe@GO's electrochemical stability was assessed in KOH electrolyte with a continuous overpotential of 0.4 V as given Fig. S2. Excellent catalytic endurance under alkaline conditions was indicated by the chrono-amperometric data, which showed a consistent current response during continuous electrolysis. The significant synergistic interaction between Ru, NiSnSe, and graphene oxide, which enhances charge transfer and structural integrity, is the source of the improved stability. Additionally, the GO structure inhibits nanoparticle aggregation and offers effective electron transport. These findings support Ru-doped NiSnSe@GO's promise as a very stable electro-catalyst for alkaline water-splitting applications.

## 4 Conclusions

This research illustrates that the modulation of electronic structure induced by doped Ru, significantly improves the bi-functional electro-catalytic performance of NiSnSe@GO for both the OER and HER under alkaline conditions. The Ru-NiSnSe@GO nanocomposites demonstrate a uniform distribution of Ru and robust interfacial bonding with graphene oxide (GO), resulting in enhanced electronic conductivity and structural characteristics. A distinct trend dependent on the dopant concentration was noted, with 1% Ru-NiSnSe@GO exhibiting the best performance, as indicated by lower over-potentials and Tafel slopes. X-ray diffraction (XRD) and Raman spectroscopy analyses confirmed lattice contraction and electronic interactions, emphasizing the role of Ru in adjusting the electronic environment of the catalyst. An increased electrochemically active surface area and reduced charge-transfer resistance further facilitated improved kinetics and charge transport. Overall, the synergistic effect of Ru doping and GO support presents a viable approach for the development of efficient and cost-effective bi-functional electro-catalysts, offering a promising pathway forward.

## Conflicts of interest

There are no conflicts to declare.

## Supplementary Material

RA-OLF-D6RA02938H-s001

## Data Availability

Data will be made available on request. Supplementary information (SI) is available. See DOI: https://doi.org/10.1039/d6ra02938h.
