# Supplementary material for: Ru-induced electronic structure modulation of NiSnSe@GO boosted oxygen and hydrogen evolution reaction under alkaline conditions
Source: RSC Adv. 2026 Jul 3;16(35):35285–98. doi: 10.1039/d6ra02938h (PMC13329754; doi:10.1039/d6ra02938h)
Supplement: RA-016-D6RA02938H-s001 [file RA-016-D6RA02938H-s001.pdf]

Supplementary Information (SI) for RSC Advances.
This journal is © The Royal Society of Chemistry 2026

Ru-induced Electronic Structure Modulation of NiSnSe@GO as Boosted Oxygen and Hydrogen Evolution Reaction Under Alkaline Conditions

Afifah Jabeen[1], Arif Nazir*[1], Shahid Bashir[2], Munawar Iqbal[3, 4], Muhammad Owais[5], Hassan Tariq[5], Murat Kaleli[6], Salih Akyürekli[6], Abid Ali[1]

[1]Department of Chemistry, The University of Lahore, Lahore, Pakistan
[2]Power Energy Dedicated Advanced Centre, Universiti Malaya, 50603, Kuala Lampur, Malaysia
[3]Zero Emission Technologies Innovation Center, University of Tabuk, Tabuk, 47913, Saudi Arabia
[4]Department of Chemistry, Faculty of Science, University of Tabuk, Tabuk, 47913, Saudi Arabia
[5]Flexible Electronic Lab, Department of Physics, International Islamic University, Islamabad, Pakistan
[6]Innovative Technologies Application and Research Center (YETEM), Süleyman Demirel University, Isparta 32260, Turkey

***Corresponding Author Email:** anmalik77@gmail.com

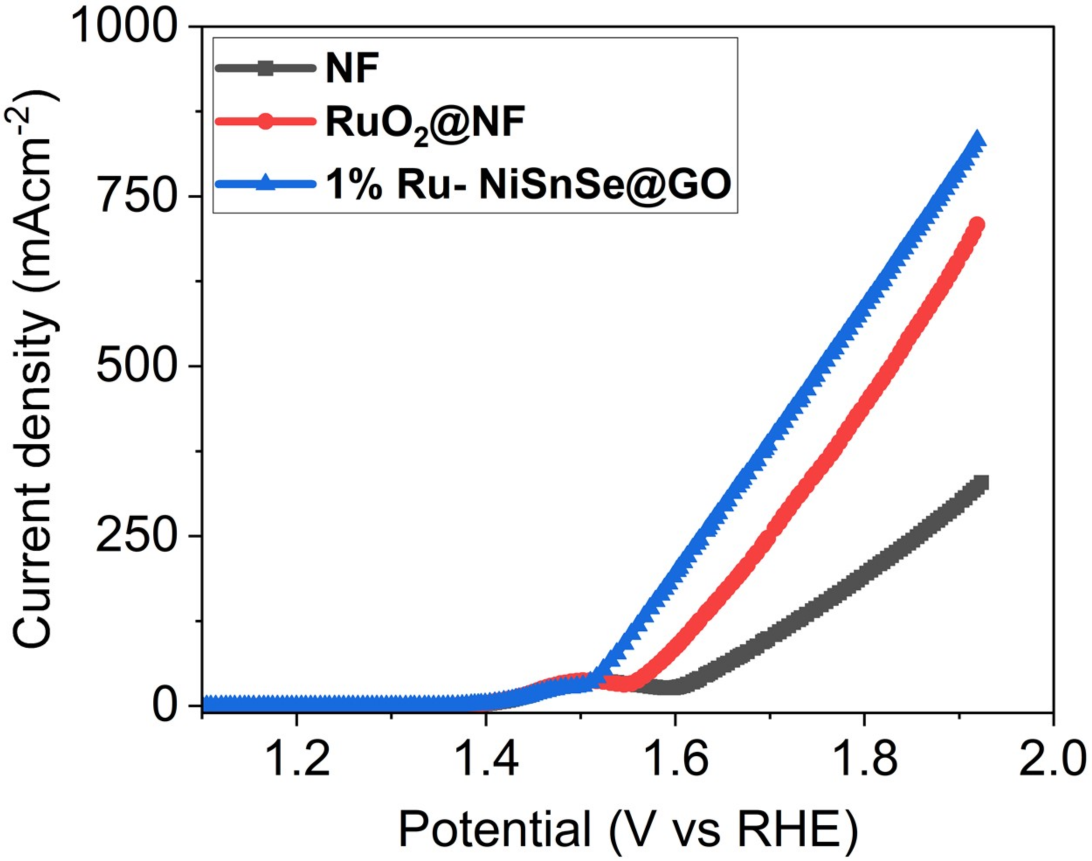

Fig. S1. LSV curves for Ni-foam RuO2 modified NF and their comparison with the as fabricated electrode in oxygen evolution reactions under alkaline conditions.

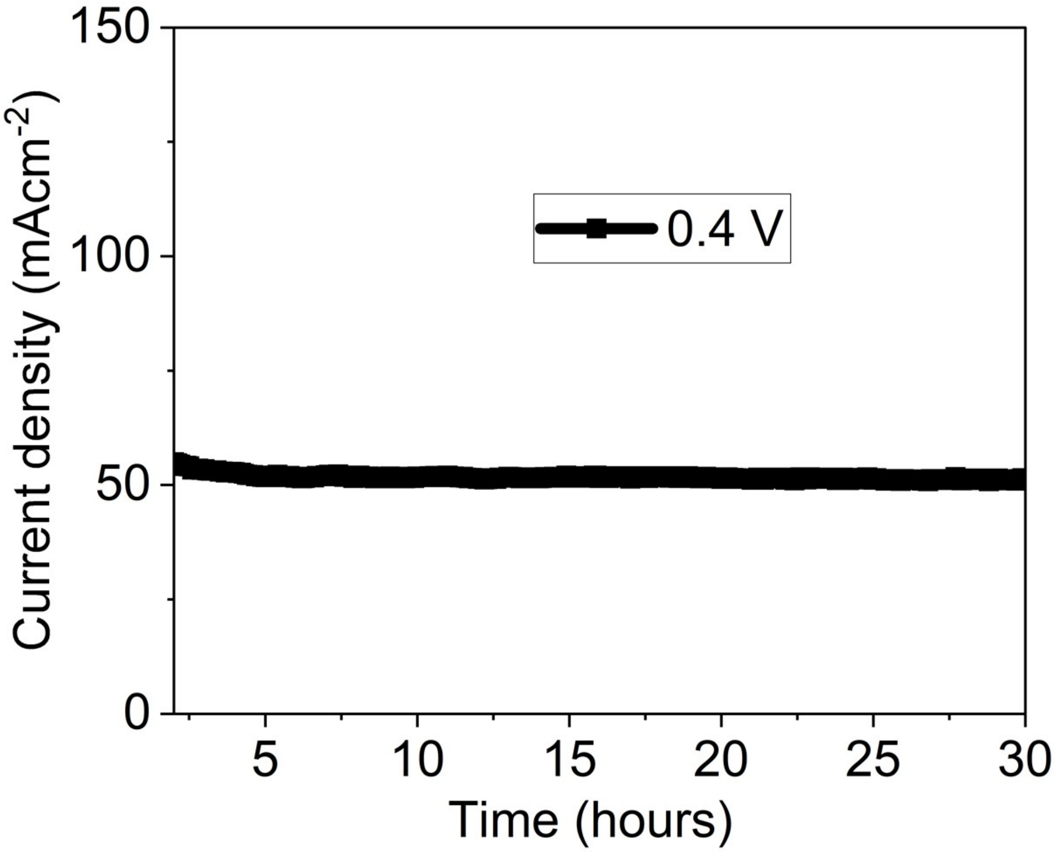

Fig. S2. Chronoamperometric curves of OER at constant overpotentials of 0.4 V under alkaline conditions.
